# Protective efficacy of the chimeric *Staphylococcus aureus* vaccine candidate IC in sepsis and pneumonia models

**DOI:** 10.1038/srep20929

**Published:** 2016-02-11

**Authors:** Liuyang Yang, Changzhi Cai, Qiang Feng, Yun Shi, Qianfei Zuo, Huijie Yang, Haiming Jing, Chao Wei, Yuan Zhuang, Quanming Zou, Hao Zeng

**Affiliations:** 1 National Engineering Research Center of Immunological Products & Department of Microbiology and Biochemical Pharmacy, College of Pharmacy, Third Military Medical University, Chongqing 400038, P.R. China; 2 Department of Biological and Chemical Engineering, Chongqing University of Education, Chongqing 400067, P.R. China

## Abstract

*Staphylococcus aureus* causes serious sepsis and necrotic pneumonia worldwide. Due to the spread of multidrug-resistant strains, developing an effective vaccine is the most promising method for combating *S. aureus* infection. In this study, based on the immune-dominant areas of the iron surface determinant B (IsdB) and clumping factor A (ClfA), we designed the novel chimeric vaccine IsdB_151-277_ClfA_33-213_ (IC). IC formulated with the AlPO_4_ adjuvant induced higher protection in an *S. aureus* sepsis model compared with the single components alone and showed broad immune protection against several clinical *S. aureus* isolates. Immunisation with IC induced strong antibody responses. The protective effect of antibodies was demonstrated through the opsonophagocytic assay (OPA) and passive immunisation experiment. Moreover, this new chimeric vaccine induced Th1/Th17-skewed cellular immune responses based on cytokine profiles and CD4^+^ T cell stimulation tests. Neutralisation of IL-17A alone (but not IFN-γ) resulted in a significant decrease in vaccine immune protection. Finally, we found that IC showed protective efficacy in a pneumonia model. Taken together, these data provide evidence that IC is a potentially promising vaccine candidate for combating *S. aureus* sepsis and pneumonia.

*Staphylococcus aureus* is a major bacterial pathogen that causes a variety of community- and hospital-acquired diseases, such as skin infections, bacteraemia, pneumonia, and endocarditis^1^,^2^. In the United States, methicillin-resistant *S. aureus* (MRSA) strains are responsible for approximately 50% of nosocomial staphylococcal infections, and multidrug-resistant isolates are becoming more and more common^2^. The emergence of multidrug-resistant strains, including some strains that are resistant to vancomycin^3^,^4^,^5^, underscores the need to develop new strategies, such as vaccines, to combat the spread of *S. aureus*.

To date, several *S. aureus* vaccine trials based on active or passive immunisations have been attempted^6^,^7^,^8^, but no clinical trials have succeeded. Several interpretations of these failed trials have been offered: 1) one antigen may not be sufficient to protect humans from *S. aureus* infections; 2) the levels of antibodies were measured, but the levels of functional antibodies were not specified; and 3) the humoral immunity alone cannot protect humans against *S. aureus* infections^9^. Recent research has suggested that vaccines that can induce cellular responses are more advantageous compared with vaccines based on humoral responses alone^10^. Indeed, combining appropriate antigens that can stimulate both humoral and cellular responses is the strategy most likely to generate protective immune responses against *S. aureus* infection^11^.

Surface proteins are crucial for *S. aureus* colonisation and virulence. Therefore, recombinant cell wall-anchored antigens have been proposed as potential *S. aureus* vaccine candidates^12^. The IsdB belongs to the near iron transporter (NEAT) motif family and consists of two domains, NEAT1 and NEAT2, which bind haemoglobin and remove heme, respectively^13^. Immunisation with IsdB yielded good protective effects in a murine sepsis model that seemed to correlate with anti-IsdB antibody titres^14^. Adoptive transfer of antigen-specific Th17 cells induced by immunisation with IsdB also conferred protection in murine models^14^,^15^.

ClfA is a fibrinogen-binding microbial surface component that recognises adhesive matrix molecules, and almost all *S. aureus* strains express this antigen^16^. It plays a critical role in the binding of *S. aureus* to fibrinogen^17^ and promotes *S. aureus* adhesion to blood clots^18^, biomaterial surfaces^19^, and damaged endothelial surfaces^18^. ClfA also mediates pathogen binding to platelets in a catheter-induced staphylococcal endocarditis model^20^. The functional domain A, which plays a fibrinogen-binding role, is comprised of 520 residues (ClfA_40-559_). The second domain is the region R, which is composed of serine-aspartate dipeptide repeats. Immunisation with ClfA_40–559_ induced strong antibody responses and showed a protective effect in a septic arthritis model^16^. In other studies, passive immunisation with antibodies targeting ClfA_40–559_ mediated protection in animals^21^,^22^. Furthermore, immunisation with ClfA induced IL-17A immune responses, which were shown to be protective against *S. aureus* infection^23^.

IsdB and ClfA have both been proposed as potential vaccine targets^9^. However, clinical trials based on these two candidates have failed for unpublished reasons^24^,^25^. Despite these failures, both IsdB and ClfA can induce strong humoral and cellular immune responses in animal models. Moreover, they are conserved across *S. aureus* strains^1^. Thus, these two candidates are still considered highly attractive molecules for vaccine development. It is possible that one antigen may not be sufficient to induce ideal immune protection; therefore, a vaccine that targets several surface proteins may exhibit increased efficacy^12^. For example, a vaccine that combined four surface proteins induced a more ideal protective effect in mice^26^ than vaccines that targeted each protein individually. This finding supports the hypothesis that multivalent antigens in vaccines may be more likely to induce ideal protection in future clinical trials^27^,^28^,^29^.

In this report, we selected IsdB and ClfA as our candidate antigens. We combined the NEAT1 domain of IsdB and the fibrinogen-binding domain of ClfA to generate the novel chimeric vaccine IsdB_151-277_ClfA_33-213_ (IC). We demonstrated that immunisation with IC could elicit strong antibody responses that promoted opsonophagocytic killing *in vitro* and conferred protection *in vivo* against *S. aureus* sepsis. The IL-17A immune pathway played an important role in IC immune protection. In support of a growing consensus that multiple antigenic targets may be required to formulate an effective anti-staphylococcal vaccine, our data suggest that IC is a potentially promising *S. aureus* vaccine component.

## Materials and Methods

### Ethics statement

All animal care and use protocols in this study were performed in accordance with the Regulations for the Administration of Affairs Concerning Experimental Animals approved by the State Council of People’s Republic of China. All of the animal experiments were approved by the Animal Ethical and Experimental Committee of the Third Military Medical University (Chongqing, Permit No. 2011-04). All surgery was carried out under sodium pentobarbital anesthesia, and all efforts were made to minimize suffering.

### Animals and bacterial strains

BALB/c and C57BL/6 mice (7- to 8-week-old females) were purchased from Beijing HFK Bioscience Limited Company (Beijing, People’s Republic of China). IL-17A gene knockout (IL-17AKO) mice (C57BL/6 background) were kindly provided by Richard A. Flavell (Yale University School of Medicine, New Haven, CT, USA). Female New Zealand white rabbits (weighing 2.00 ± 0.20 kg) were provided by TengXin Company (Chongqing, People’s Republic of China). The animals were maintained under specific-pathogen-free (SPF) conditions. *S. aureus* strain MRSA 252 was purchased from the American Type Culture Collection (Manassas, VA, USA). The WHO2 strain was provided by Hong Zhou (Third Military Medical University, China). The three other clinical *S. aureus* isolates were collected from three different hospitals in China (Supplemental Table 1). The bacteria were cultured in tryptic soy broth at 37 °C for 6 h, centrifuged at 5000 × g for 5 min, washed with phosphate-buffered saline (PBS) 3 times, and diluted with PBS to an appropriate cell concentration using spectrophotometry at 600 nm.

### PCR amplification

The complete genome of strain MRSA 252 was used as the PCR template. IsdB_35-629_ was generated in our previous work^30^. The ClfA_33-213_ gene was amplified by PCR primers P3 and P4 (Supplemental Table 2). For the IsdB_151-277_-TACGTTCCGGTTGATGTT-ClfA_33-213_ construct, the first round of PCR was performed using the primers P1 and P2 (Supplemental Table 2) to generate IsdB_151-277_-TACGTTCCGGTTGATGTT. Similarly, the primers P3 and P4 were used to generate TACGTTCCGGTTGATGTT-ClfA_33-213_. By employing the first-round PCR products as the templates, the second round of PCR was performed using the primers P1 and P4. BamHI and NotI sites were introduced at the beginning and end of the PCR products through the primers to obtain the amplified genes.

### Expression and purification of recombinant proteins

Briefly, the PCR product was cloned into pGEX-6p-2 (Sangon Biotech, shanghai) and transformed into the *Escherichia coli* strain Xl1-blue (Biovector Scienc Lab, Beijing). *Escherichia coli* strain Xl1-blue containing the recombinant plasmid was induced with isopropyl-b-D-1-thiogalactopyranoside (IPTG) at a final concentration of 0.1 mM for GST fusion protein expression. We purified the GST-tagged proteins from the cleared lysates by Capto MMC (GE), and the GST tag was cleaved by preScission protease (GE). Then, we removed the endotoxin from the protein eluate using Triton X-114 phase separation as described elsewhere^31^. The resulting protein was analysed by gel-filtration using the Superdex^TM^ 200 10/300GL column (GE). Protein purity was determined using sodium dodecyl sulphate-polyacrylamide gel electrophoresis (SDS-PAGE) and using high-performance liquid chromatography (HPLC) with a C3 column. The concentration of the resulting protein was determined using the bicinchoninic acid (BCA) method (Pierce). The endotoxin content was detected using the tachyplens ameboyto lysate assay (Houshiji cod Inc., Xiamen, China).

### Immunisation of the mice and *S. aureus* infection

For active immunisation, purified proteins were dissolved in PBS and emulsified 1:1 (volume ratio) in AlPO_4_ (Pierce), Al(OH)_3_ (Pierce), or Freund’s adjuvant (Sigma). Mice were intramuscularly injected with 100 μL of the emulsion containing 20 μg protein, isovolumetric PBS plus adjuvant, or PBS alone as the control on days 0, 14, and 21. On day 28, the mice were exsanguinated, and serum samples were collected for the enzyme-linked immunosorbent assay (ELISA).

To measure the survival rates, immunised BALB/c mice were intravenously infected with MRSA 252 [1 × 10^9^ colony-forming units (CFUs)], WHO2 (5 × 10^8^ CFUs), or the clinical *S. aureus* isolates (3 × 10^8^ CFUs) on day 35 and monitored for survival for 14 days after infection. For bacterial burdens and histopathology analyses in the sepsis model, an infective dose (5 × 10^8^ CFUs) of MRSA 252 was intravenously administered in each mouse.

For the pneumonia model, C57BL/6 mice were anaesthetised with isoflurane (RWD Life Science) and inoculated with 4 × 10^8^ CFUs of a MRSA 252 suspension in the naris. Then, the mice were monitored for 7 days after infection. Thermalert TH-5 (Physitemp) was used for determination of the intrarectal temperature of infected mice.

### Quantitative bacteriology in organs

Lungs, spleens, and kidneys from corresponding animals were removed, weighed, and homogenised in 2 mL of PBS. Peripheral blood was collected in heparin anticoagulant tubes. All samples used for quantitative cultures were grown on tryptic soy broth for 24 hours at 37 °C.

### Histological analysis

Kidney tissues were obtained 3 days post-infection from the sepsis model mice, and lung tissues were collected 1, 3, and 7 days post-infection from the pneumonia model mice. The organs were fixed with 10% formalin and embedded in paraffin.

A double-blind histological analysis was performed to determine the severity of each section of lung or kidney. Each lung section from the pneumonia model mice was given a score of 0–4 (no abnormality to most severe) according to established criteria^32^. Each kidney section from the sepsis model mice was given a score of 0–4 (0: no abnormality; 1: area of renal tubular interstitial lesion <5%; 2: 5%–25%; 3: 25%–75%; and 4: > 75%).

### ELISA for specific antibodies

The ELISA was performed as previously described^30^. Serum samples were used as the primary antibodies. The secondary antibodies were HRP-conjugated goat anti-mouse IgG (Southern Biotech, Birmingham, AL, USA), anti-IgG1, or anti-IgG2a (Sigma). The titres were diluted and monitored at OD450 or defined as the highest dilution that yielded an absorbance value of more than twice the value of the blank control.

### Opsonophagocytic assay

The assay was performed based on a method described by Burton and Nahm^33^. Briefly, MRSA 252 was used as the target strain, and the HL60 cell line (ATCC, CCL-240) was used as the opsonophagocytic cells. HL60 cells were maintained in L-glutamine-containing IMDM medium (HyClone) supplemented with 20% heat-inactivated FBS (HyClone), 100 U/mL penicillin, 100 μg/mL streptomycin, and 0.25 μg/mL amphotericin (Mediatech) at 37 °C in 5% CO_2_. To differentiate the granulocytes, HL60 cells (5 × 10^5 ^cells/mL) were cultured in medium with 0.8% N,N-dimethylformamide (Sigma) for 4 days. Trypan blue tests were performed to verify HL60 cell viability, and results ≥90% were considered acceptable for the opsonophagocytic assay (OPA). The assay was performed in 96-well round-bottom plates. Each well (volume, 80 μL) contained 4 × 10^5^ HL60 cells, 10^3^ CFUs of MRSA 252, vaccinated rabbit antisera, and 3- to 4-week-old infant rabbit serum as a complement source. Then, the microtitre plates were shaken on a mini-orbital shaker (700 rpm) for 45 minutes in an incubator (37 °C with 5% CO_2_). For termination, the microtitre plates were placed on ice for 20 minutes. Next, 20 μL of the reaction mixture from each well was spotted onto tryptic soy broth. The samples were plated in duplicate, and the killing effect was defined as a reduction in CFUs after overnight growth. Control samples were incubated with normal rabbit serum (NRS). The percentage of opsonophagocytic killing was determined by subtracting the number of colonies that survived the test assay from the number of CFUs on the NRS control.

### Passive immunisation

To generate rabbit polyclonal antibodies, an immunisation procedure was performed on rabbits similar to the procedure performed on mice. On day 28, the serum was pooled and the IgG fraction was obtained. The IgG concentration was determined by the BCA method. One day before infection, 5 mg of antibody per mouse was passively immunised via the intraperitoneal (i.p.) route (n = 10). Survival was monitored for 12 days.

### Splenocyte stimulation test

The amounts of IFN-γ and IL-17A in the cell culture supernatants were determined based on a previously described method^30^. Spleens were aseptically removed, and cells were suspended at a concentration of 2 × 10^6^ cells/mL in complete media (RPMI 1640 with 10% FBS). The cells were stimulated with or without 10 μg/mL of IsdB, ClfA_33-213_, or IC protein at 37 °C for 5 days. The supernatants were collected, and the amounts of IFN-γ and IL-17A were determined by ELISA using mouse IFN-γ ELISA and IL-17A ELISA kits (Biolegend), respectively.

### Flow cytometry to determine the percentage of Th1/Th17 cells

After stimulation for 5 days, splenocytes were harvested and stimulated with PMA (Sigma, 50 ng/mL), ionomycin (Sigma, 1 mM), and Golgistop (BD Biosciences, 2μl in 3mL culture medium) for another 6 h. Then, the splenocytes were incubated with FITC-anti-mouse CD3 (GK1.5), APC-anti-mouse CD4 (GK1.5), PE-anti-mouse IFN-γ (XMG1.2), and PerCP-Cyanine5.5-anti-mouse/rat IL-17A (eBio17B7) (eBioscience) in FACS buffer (1% BSA, 1% duck serum, and 0.01% sodium azide) at 4 °C for 30 min. The cells were then washed 3 times with PBS. The percentage of Th1 (IFN-γ^+^ IL-17A^−^) and Th17 (IFN-γ^−^ IL-17A^+^) cells was evaluated using a FACSCalibur (BD Biosciences) and analysed using the CellQuest Pro software (BD Biosciences).

### Inhibition of the IFN-γ and/or IL-17A immune pathways

Monoclonal antibodies were applied to the vaccinated group to induce the functional inhibition of IFN-γ or IL-17A. Purified monoclonal anti-mouse IFN-γ (αIFN-γ; eBioscience; 16-7311-85) or anti-mouse IL-17A (αIL-17A; eBioscience; 16-7173-85) was prepared using endotoxin-free PBS. Two days before infection, vaccinated mice were injected with 100 μg (i.p.; 100 μL) of αIFN-γ, αIL-17A, or both. The vaccinated and nonvaccinated control groups were injected with the nonspecific isotype control IgG [100 or 200 μg (i.p.)] at doses comparable to those administered to the single inhibition group and the combined treatment group.

### Statistical analysis

Data are presented as the means ± SD or means ± SEM. The non-parametric log rank test was used for determining the differences in the survival rates. The Student’s t-test was used for determining the antibody titers. Otherwise, the non-parametric Mann–Whitney test was used for analysis the bacterial burdens and the severity score of kidneys and lungs. GraphPad Prism 5.0 (GraphPad Software) was used for data analyses, and a p-value < 0.05 was considered significant.

## Results

### Expression and purification of IsdB, ClfA_33-213_, and IC

**Figure 1:**
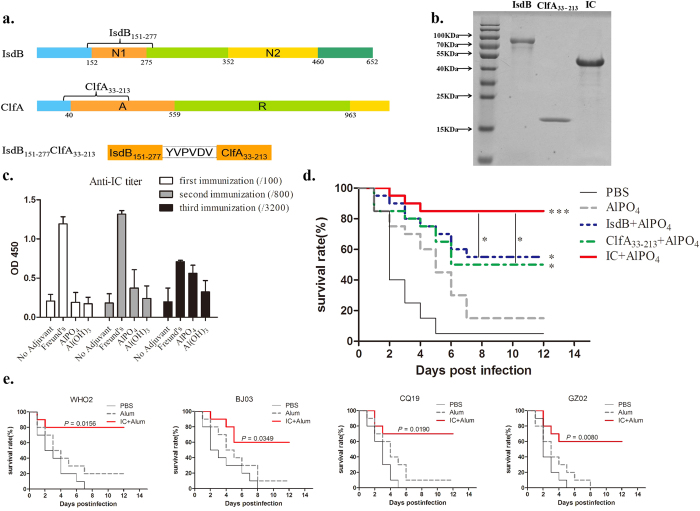
Cloning and expression of the recombinant protein IsdB_151-277_ClfA_33-213_ (IC), and validation the protection effects in lethal *S. aureus* sepsis model. (**a**) Schematic diagram illustrating the primary structure of the IsdB_151-277_, ClfA_33-213_ and IsdB_151-277_ClfA_33-213_ (IC). (**b**) Recombinant structure of IsdB, ClfA_33-213_ and IC were purified by affinity chromatography and analysed by SDS-PAGE. (**c**) Antibody responses were expressed as mean absorbance at 450 nm ± SD. BALB/c mice (n = 6) were immunised at day 0, 14 and 21. Serum was taken three times at day 7, 21 and 28, and the absorbance was monitored at dilution of 1:100, 1:800, and 1:3200, respectively. (**d**) BALB/c mice (n = 20) were immunised with antigens plus AlPO_4_ adjuvant. The mice were intravenously infected with MRSA 252 (1 × 10^9^ CFUs), and the survival rates were monitored for 12 days. (**e**) BALB/c mice (n = 10) were challenged by an intravenous injection of WHO2 (5 × 10^8^ CFUs) and clinical *S. aureus* isolates (3 × 10^8^ CFUs) after immunisation. Survival rates were monitored for 12 days. Compared with animals receiving the AlPO_4_, the significance of the protective immunity generated by the various antigens was measured with a log rank test. The asterisks represent a statistically significant difference (^*^P < 0.05, ^**^P < 0.01, ^***^P < 0.001).

We amplified IsdB_151-277_ and ClfA_33-213_ by PCR and combined them using the “YVPVDV” linker (Fig. 1a). Recombinant ClfA_33-213_ and IC were expressed in *Escherichia coli* strain Xl1-blue in the soluble fraction. We purified IsdB_35-629_ in our previous work^30^; this peptide was used as the control for the individual IsdB component in this study. As demonstrated by SDS-PAGE in Fig. 1b, the masses of the recombinant proteins IsdB, ClfA_33-213_, and IC were in accordance with their predicted molecular masses (98, 19, and 36 kDa, respectively).

### Validation of the optimal adjuvant and protective effect of IC in a lethal *S. aureus* sepsis model

To choose an optimal adjuvant for the IC immunisation strategy, we immunised mice with the same dose of IC plus different adjuvants (Freund’s adjuvant, AlPO_4_, and Al(OH)_3_). The Freund’s adjuvant group showed significantly high titres in the serum antibody detection test after the first two immunisations; however, after the third boost, AlPO_4_ induced titres nearly the same as those detected for Freund’s adjuvant (Fig. 1c). Because Freund’s adjuvant is not recommended for use in humans due to its side effects, we chose AlPO_4_ as the optimal adjuvant in the following experiments. Mice were immunised with IsdB, ClfA_33-213_, and IC plus AlPO_4_ as the adjuvant, and the protective effects against *S. aureus* infection were evaluated in a sepsis model. The animals vaccinated with the IsdB, ClfA_33-213_, or IC antigens exhibited higher survival rates (55%, 50%, and 85% 12 days post-infection, respectively) compared with the AlPO_4_ group (15% survival) and the PBS group (5% survival). The significance of the protective effects compared with those of AlPO_4_ was assessed using a log rank test (*P*_*IsdB*_ = 0.0151; *P*_*ClfA33-213*_ = 0.0423; and *P*_*IC*_ < 0.0001). The chimeric vaccine yielded a higher protective effect than IsdB or ClfA_33-213_ alone (Fig. 1d, *P*_*IC-IsdB*_ = 0.0498 and *P*_*IC-ClfA33-213*_ = 0.0260). Therefore, vaccination with IC can generate a relatively ideal protective effect in a lethal *S. aureus* sepsis model.

We used several *S. aureus* strains to test the broadness of the protective effect against *S. aureus*. We collected several clinical *S. aureus* isolates from different hospitals throughout China (Beijing, Guangzhou, and Chongqing), and all three of the isolates expressed both IsdB and ClfA (Supplementary Table S1). Then, we validated the protective effects against WHO2 and the three clinical *S. aureus* isolates. As shown in Fig. 1e, immunisation with IC induced a broad protective effect against WHO2 and the three clinical isolates. These data indicated that this novel vaccine yielded broad protective effects against several *S. aureus* strains in a sepsis model.

### Immunisation with IC significantly reduced the bacterial burdens in different organs and decreased pathological damage to the kidneys

**Figure 2:**
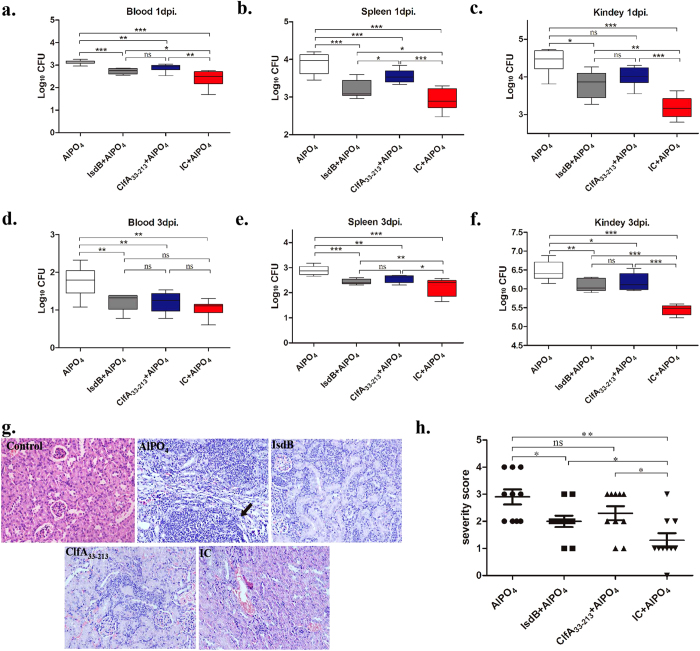
Immunisation with IC significantly reduced both organ bacterial burdens and pathology. Bacterial burdens in the blood, spleens and kidneys (n = 8) were calculated at 1 (**a–c**) and 3 days (**d–f**) post-infection. Representative results from one of three independent experiments were shown. Data were presented as box and whisters, and the medians were shown. Asterisks indicate significant differences between two groups (^*^P < 0.05, ^**^P < 0.01, ^***^P < 0.001). (**g**) HE-stained kidneys from the AlPO_4_ group and recombinant vaccine immunised groups at 3 days post-infection were shown. BALB/c mice were immunised with recombinant proteins. Three days post-infection (5 × 10^8^ CFUs), the kidneys were collected, and representative histopathological sections were shown (magnification = 400×). The kidney tissue of mouse without infection were used as control. Arrowheads indicate Staphylococcal abscesses. (**h**) Severity scores of kidneys (n = 10) from the AlPO_4_ group and recombinant vaccine immunised groups at 3 days post-infection were shown. Data were presented as scatter plots, and the means ± SEM were shown. Asterisks indicate significant differences between two groups (^*^P < 0.05, ^**^P < 0.01).

The bacterial burdens in the blood, spleens, and kidneys were calculated 1 and 3 days post-infection. In the blood, the bacterial burdens were much lower in the IC group 1 day post-infection compared with IsdB (*P* = 0.0370) and ClfA_33-213_ (*P* = 0.0060) (Fig. 2a). However, there was no significant difference in these three vaccinated groups 3 days post-infection (Fig. 2d). In the kidneys and spleens, the elimination of *S. aureus* was enhanced in the IC group within 3 days post-infection compared with the IsdB and ClfA groups (Fig. 2a–f). These results showed that immunisation with IC protected against *S. aureus* infection by reducing *S. aureus* colonisation in organs and preventing a direct attack on the organs by the bacteria. Consistent with the survival rates, immunisation with IC induced a higher protective effect against *S. aureus* by reducing the bacterial burden.

Based on our histological analysis, the kidneys from the mice in the recombinant vaccine immunised groups showed less inflammatory cell infiltration and renal abscesses after infection compared with the AlPO_4_ group (Fig. 2g). Furthermore, the glomeruli in the IC group showed less damage, with fewer inflammatory cells detected in the kidneys. In contrast, the kidneys in the AlPO_4_ group harboured bacterial abscesses with several large foci of staphylococci (Fig. 2g). Taken together, the results demonstrate that the severity of the kidney damage was significantly lower in the IC group than in the IsdB and ClfA_33-213_ groups (Fig. 2h).

### Active immunisation with IC induced strong antibody responses

Next, we determined the antigen-specific antibody responses in the sera. The recombinant proteins plus AlPO_4_ produced strong antibody responses. The total titres of IgG and the IgG subgroups (IgG1 and IgG2a) in the IC-immunised mouse group were much higher compared with those in the mice immunised with IsdB or ClfA_33-213_ (Table 1).

To determine whether antibodies were generated against both components in the IC-immunised group, we analysed antigen-specific IgG in the sera of the IC group (Table 2). There were no significant differences in the levels of antibodies targeting IsdB and ClfA induced by IC compared with those generated by immunisation with the single components alone. These results show that immunisation with IC alone can generate strong antibody responses against both IsdB and ClfA.

### Opsonophagocytic assay and passive immunisation test corroborated the importance of antibodies in vaccine protective efficacy

**Figure 3:**
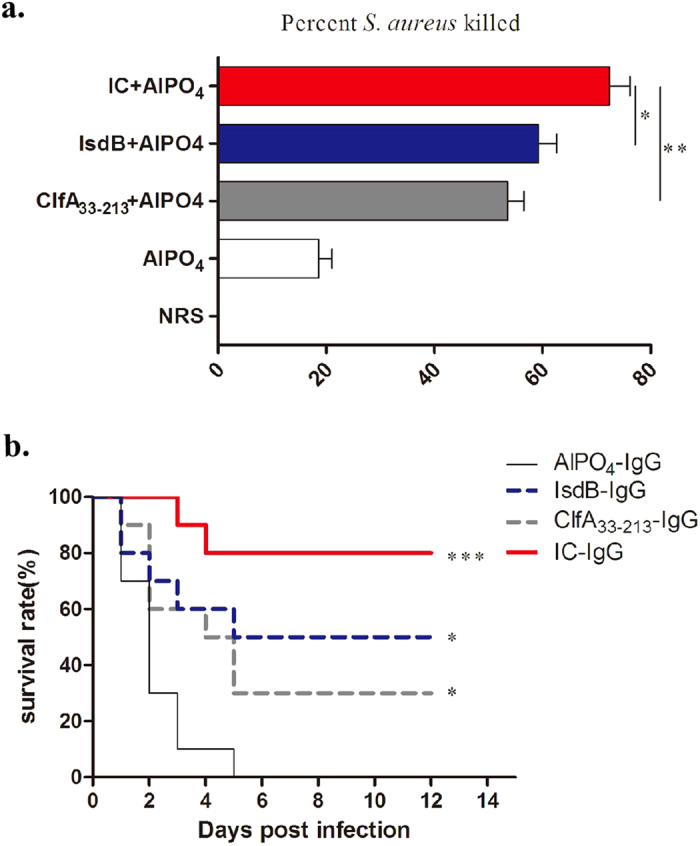
Opsonophagocytic assays and passive immunisation. Rabbits (n = 6) were immunised with AlPO_4_, IsdB, ClfA_33-213_ and IC, serum was separated. (**a**) The results of opsonophagocytic assays were shown. Rabbit serum (1:5 diluted), fetal rabbit complement, HL60 cells, and MRSA 252 were incubated in round bottom 96-well plates with shaking, and plated on tryptic soy broth to measure bacterial survival counted by CFUs, then the percent of killing was calculated. Normal rabbit serum was used as blank control. Error bars represent the SD. (**b**) Survival rates of passive immunisation were shown. Antibodies specific IgG in the rabbit serum was purified. One day before infection, BALB/c mice (n = 10) were passively immunised intraperitoneally (i.p.) with 5 mg of the IgG fraction containing antibodies specific for IC, IsdB, ClfA_33-213_ and AlPO_4_, respectively. Mice were challenged with lethal dose of MRSA 252 (1 × 10^9^ CFUs), survival rates were monitored for 12 days. The significance of the protective immunity generated by the various antigens was measured with a log rank test compared with AlPO_4_ group. The asterisks represent a statistically significant difference (^*^P < 0.05, ^**^P < 0.01, ^***^P < 0.001).

To evaluate the efficacy of the IC-specific antibodies, an OPA that measured antibody- and complement-mediated bacterial killing was performed *in vitro*. The killing assay was performed using immune cells (neutrophils) that play a critical role in the host clearance of *S. aureus*. In the presence of HL60 phagocytic cells and complement, rabbit serum raised against the three proteins exhibited opsonophagocytic activity against *S. aureus*. As shown in Fig. 3a, the percentages of *S. aureus* killed in the IC group (70.1%), IsdB group (56.3%), and ClfA_33-213_ group (51.8%) were higher than those killed in the AlPO_4_ group (17.5%). The IC-specific antibodies were more efficient than the IsdB-specific (*P* = 0.0300) and ClfA_33-213_-specific antibodies alone (*P* = 0.0035). These results indicated that the IC-specific antibodies were more effective in killing *S. aureus*; thus, the antibody response may functionally regulate phagocytosis and killing of *S. aureus* by innate immune cells.

We confirmed that IC could induce functional antibodies that were effective *in vitro*. However, whether these antigen-specific proteins provided protective immunity *in vivo* remained unclear. To verify the protective role of antibodies *in vivo*, we performed passive immunisation. The mice passively immunised with IC showed higher protection (80% survival) compared with the IsdB (50% survival), ClfA_33-213_ (30% survival), and AlPO_4_ IgG-treated groups (0% survival) (Fig. 3b). Anti-IC antibodies showed higher protective effects compared with those in the ClfA_33-213_ group and IsdB group. We conclude that anti-IC antibodies effectively protect mice against *S. aureus* infection.

### Cytokine responses and CD4^+^ T cell polarisation of splenocytes

**Figure 4:**
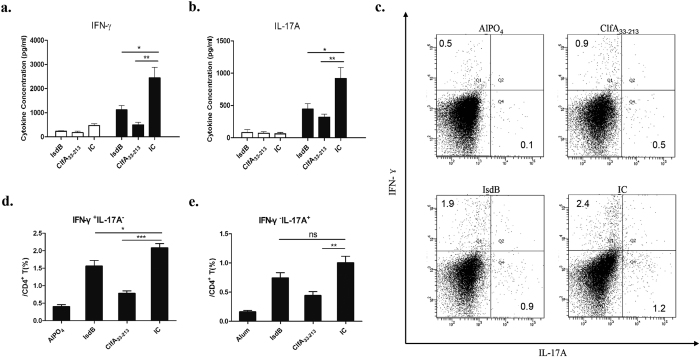
Cytokine responses and CD4^+^ T cell polarization of splenocytes. One week following the last booster, the splenocytes from vaccinated mice (n = 6) were incubated with corresponding antigen proteins (10 μg/mL) for 5 days. The supernatants were harvested, and the cytokine levels of (**a**) IFN-γ, (**b**) IL-17A were determined. The means ± SEM were shown. Empty boxes representative without stimulation, black boxes representative splenocytes stimulate with corresponding proteins. Asterisks indicate significant differences. (^*^P < 0.05, ^**^P < 0.01). (**c**) After 5 days incubation, the splenocytes were stimulated with PMA/ionomycin and Golgistop for 6 h. Cells were stained for CD3, CD4, IFN-γ, or IL-17, and was analysed by flow cytometry. The responses of Th1 (**d**) and Th17 (**e**) of the AlPO_4_ group and recombinant vaccine immunised groups were presented as column bar graph and shown as means ± SEM. Results represent three independent experiments (^*^P < 0.05, ^**^P < 0.01, ^***^P < 0.001).

Next, we evaluated antigen-specific cellular responses. The amounts of cytokines in the supernatants were measured after stimulation with recombinant proteins. Splenocytes from the vaccinated mice (IsdB, ClfA_33-213_, and IC) produced significantly more IFN-γ and IL-17A (Fig. 4a,b) when stimulated with the corresponding recombinant proteins than splenocytes from the AlPO_4_ group. Furthermore, the splenocytes from the IC group produced the highest amount (Fig. 4a,b, *P* < 0.05), which showed that splenocytes from mice immunised with IC could produce more IFN-γ and IL-17A following antigen encounters.

Then, the splenocytes were harvested for T cell polarisation tests *in vitro*. After stimulation, the splenocytes in the IC group showed an increased ability to polarise toward the production of IFN-γ and IL-17A compared with the splenocytes in the IsdB and ClfA_33-213_ groups (Fig. 4c). Although CD4^+^ T cells from mice immunised with IC were more likely to produce IL-17A than the IsdB group, this difference did not reach statistical significance (*P* = 0.1148) (Fig. 4e). The IC group had a higher number of IFN-γ^+^ IL-17A^−^ CD4^+^ T cells than the IsdB group (*P* = 0.0365) and ClfA_33-213_ group (*P* < 0.0001, Fig. 4d). These results showed that immunisation with IC increased the potential of splenocytes to induce cytokine responses and polarise toward Th1/Th17 sub-group subsets.

### Roles of IL-17A in IC vaccine efficacy

**Figure 5:**
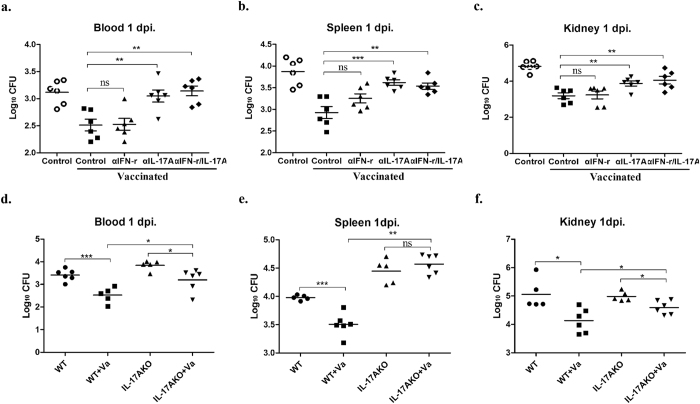
Impact of IFN-γ and/or IL-17A neutralisation on bacterial burdens in the protective effects of IC vaccination. Vaccinated BALB/c mice were injected with αIFN-γ, αIL-17A, or both at 2 days before infection. The unvaccinated mice or untreated vaccinated mice regarded as control or vaccinated control. Bacterial burdens were calculated in the blood (**a**), spleens (**b**) and kidneys (**c**) 3 days post-infection. (**d–f**) IL-17KO and WT mice were immunised as described before, the bacterial burdens were monitored 3 days post-infection. Data are presented as scatter plots, and the medians were shown. Asterisks indicate significant differences between vaccinated and control mice. Results represent three independent experiments (^*^P < 0.05, ^**^P < 0.01, ^***^P < 0.001).

To evaluate the potential roles of IFN-γ and IL-17A in vaccine efficacy, neutralising antibodies targeting IFN-γ (αIFN-γ) and/or IL-17A (αIL-17A) were administered to vaccinated mice. Bacterial burdens in blood, spleens, and kidneys were measured 3 days post-infection. The IC-vaccinated group displayed significantly lower bacterial colonisation versus the non-vaccinated controls. Neutralisation of IL-17A alone caused a significant increase in CFUs in the blood (*P* = 0.0017), spleens (*P* = 0.0043), and kidneys (*P* = 0.0018) in the vaccinated animals compared with the untreated vaccinated controls (Fig. 5a–c). Blocking both IFN-γ and IL-17A caused a significant increase in the mean organ bacterial burdens in the vaccinated mice compared with the control group, although the difference was not significant compared with the results of inhibition of IL-17A alone (Fig. 5a–c). In contrast, neutralisation of IFN-γ alone did not influence the bacterial burdens in the vaccinated mice (*P* > 0.05).

Furthermore, we used IL-17AKO mice to confirm the results from the IL-17A neutralisation tests. In the IL-17AKO-vaccinated group, the bacterial burdens were increased in blood (*P* = 0.0100), spleens (*P* = 0.0022), and kidneys (*P* = 0.0152) compared with those in the WT vaccinated group (Fig. 5d–f). The protective efficacy of the IC vaccine was still detected in the IL-17AKO mice because the bacterial burdens in the blood and kidneys in the IL-17AKO vaccinated group were much lower than those in the IL-17AKO unvaccinated group (Fig. 5d,f). Because IL-17A gene deficiency remarkably reduced the IC immune protective effect, we demonstrated that the strong IL-17A responses induced by IC played an important role in vaccine immune protection.

### Immunisation with IC can effectively reduce lung damage in an *S. aureus* pneumonia model

**Figure 6:**
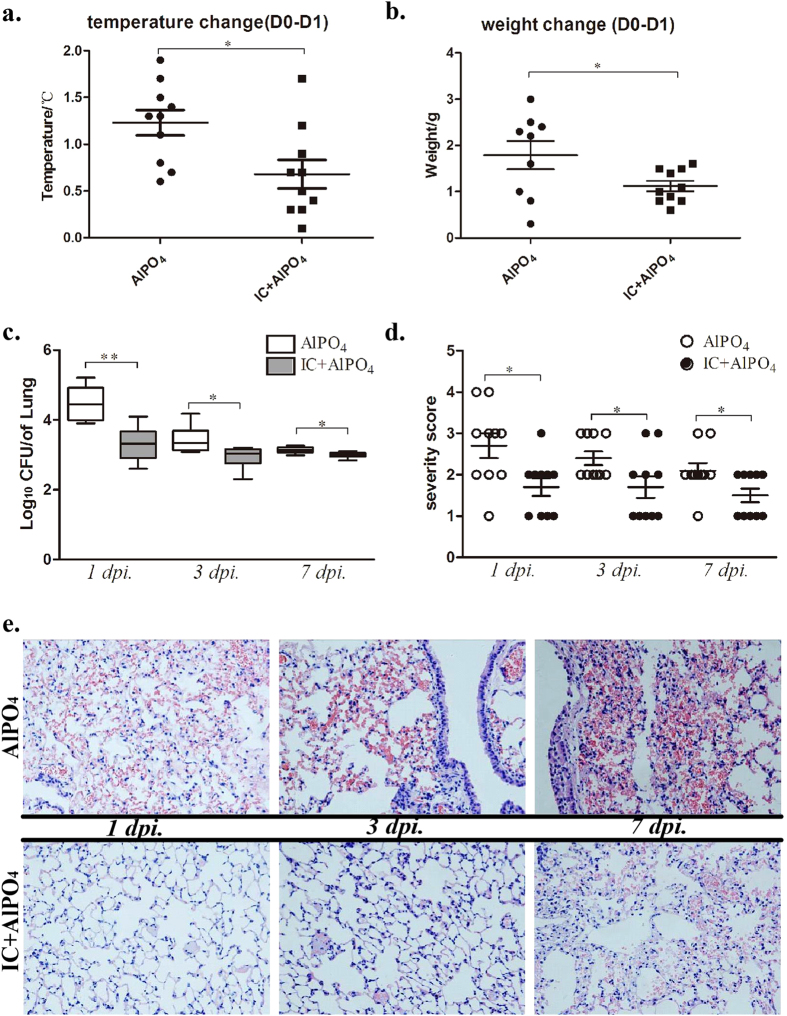
Immunisation with IC can effectively reduce lung damage in an *S. aureus* pneumonia model. C57 mice (n = 10) were immunised with IC on day 0, 14 and 21, and control group immunised with AlPO_4_. Mice were inoculated with 4 × 10^8^ CFUs of MRSA252 suspension in the naris on day 28. Temperature and weight changes were measured and shown as temperature(D0-D1) and weight(D0-D1) (**a,b**). The bacterial burdens (**c**) were calculated 1, 3 and 7 days post-infection. (**d**) The histological scores of pneumonia in four groups were monitored and presented as scatter plots. The data was presented as means derived from 3 independent experiments. (**e**) The representative HE-stained lung tissues (e, magnification = 400×) on 1, 3 and 7 days post-infection were shown.

Finally, we evaluated the IC-induced immune protective effect in *S. aureus* pneumonia. In our study, the control group immunised with AlPO_4_ alone showed serious symptoms, namely, lethargy, ruffled fur, hypothermic, and significant weight loss (Fig. 6a,b). In contrast, the body temperature and weight in the IC group were controlled within a relatively acceptable range. We calculated the bacterial burdens in the whole lung 1, 3, and 7 days post-infection and found that the bacterial burdens were significantly reduced compared with those in the AlPO_4_ group (Fig. 6c, *P*_*1day*_ = 0.0004, *P*_*3day*_ = 0.0270, and *P*_*7day*_ = 0.0095). The lung exterior also showed less haemorrhaging in the IC group (Supplementary Fig. S1), and pathological changes (Fig. 6d) and the lung severity score demonstrated that there was less damage compared with the AlPO_4_ group (Fig. 6e). Therefore, immunisation with IC significantly decreased lung damage in the *S. aureus* pneumonia model.

## Discussion

IsdB and ClfA are suitable potential candidates for use in the development of an effective *S. aureus* vaccine^12^ because almost all *S. aureus* strains express these two surface proteins^14^,^34^,^35^. Due to the increasing spread of methicillin-resistant *S. aureus* strains, it is notable that *S. aureus* generally carry these two genes. However, vaccination with IsdB and ClfA alone did not induce ideal protective effects in a murine sepsis model (Figs 1d and 2).

IsdB consists of two different domains, namely, NEAT1 and NEAT2^36^,^37^. Although the biological function of these two domains has been extensively explored, the dominant immune responses are still unknown. The NEAT1 domain was sufficient to bind haemoglobin^37^ and played a critical role in the heme-iron acquisition of *S. aureus*. Using epitope prediction, we inferred the existence of B-cell epitope enrichment regions in the NEAT1 domain. Therefore, we chose IsdB_151-277_ (NEAT1) as our vaccine candidate. Prior to cloning the extra-cellular region of ClfA_40-559_, we inferred that amino acids 33 to 213 may be suitable for the design of a vaccine using epitope prediction (www.sbc.su.se). The use of an appropriate linker peptide is necessary to retain the activity of the peptide^38^. Therefore, we designed the YVPVDV linker, which maintained the original conformation of IsdB_151-277_ and ClfA_33-213_. We linked these two immune-dominant areas together to construct the new chimeric vaccine “IC”.

The critical role of antibodies in controlling *S. aureus* infection has been reported *in vitro* and *in vivo*^39^,^40^,^41^,^42^. Notably, immunisation with IC was efficacious and induced prominent antibody responses. First, immunisation with IC induced large quantities of total IgG, IgG1, or IgG2a compared with IsdB and ClfA_33-213_ (Table 1), which showed balanced antibody production. Second, immunisation with IC alone generated similar antibody levels to those achieved by the single components (Table 2), demonstrating that this chimeric vaccine could induce both IsdB and ClfA antibodies. This finding revealed that multivalent antigens in vaccines may be more effective in combating *S. aureus*. Nevertheless, the correlation between antibody responses and immune protective effects is still unclear^39^,^43^. Using the IC group as an example, our data provided evidence that the antibody titres correlated with the protective effects.

Passively administered anti-IsdB or anti-ClfA antibodies have demonstrated efficacy in rodent models^16^. In our study, we demonstrated that polyclonal antibodies to recombinant IC were effective in preventing bacteraemia and that this prevention was superior to that afforded by IsdB or ClfA polyclonal antibodies administered alone at the same dose (Fig. 3b). This result revealed that IC-specific antibodies play an important role *in vivo* and showed that the protective effect was at least partially antibody mediated. Additionally, the opsonophagocytic properties of antibodies were assessed *in vitro*. The results showed that IC-specific antibodies could promote *S. aureus* clearance in the presence of complement, thereby facilitating neutrophil killing (Fig. 3a). The above results revealed that polyclonal antibodies to recombinant IC directly influenced protection.

An increasing amount of evidence has revealed that T cells play an important role in vaccine-mediated protective effects against pathogens^44^. Indeed, Th1 or Th17 polarised immune responses are closely related to the regulation and activation of neutrophils and macrophages^45^,^46^. In *S. aureus* infection, IL-17 mainly mediates the chemotaxis and activation of neutrophils^47^. IFN-γ is also a critical cytokine and increases the microbicidal activities of macrophages. Previous studies demonstrated that immunisation with IsdB or ClfA_40–559_ induced IL-17 production, which was critical for the vaccine protective effect against *S. aureus* infection^15^,^23^. In contrast, IsdB_N126–P361_, which contained NEAT1 and NEAT2, induced increased amounts of IFN-γ^48^. In this study, splenocytes from mice immunised with IC produced more IFN-γ and IL-17A than those from mice immunised with the single components (Fig. 4a,b). Our findings in this study were consistent with previous reports regarding the use of IsdB and ClfA in vaccine design that showed that IC played a combined role in inducing IFN-γ/IL-17A production. These results support the hypothesis that the Th1/Th17 response induced by IC may be a key cellular immune protective effect that affords protection against *S. aureus*.

Inhibition of IL-17A alone abrogated the vaccine protective effect, but neutralisation of IFN-γ alone did not influence the bacterial burdens (Fig. 5a–c). These results suggested that although IC could induce both IFN-γ and IL-17A production, the protective effect mainly depended on the Th17 pathway. Our current findings are consistent with recent reports that T cells and the Th17 pathway play critical roles in defence against *S. aureus* sepsis^9^,^10^,^49^. In IC-vaccinated mice, neutralisation of IL-17A damaged the clearance of *S. aureus* from the organs. However, the vaccinated group retained some protection despite IL-17A or IFN-γ neutralisation compared with the nonvaccinated controls. These results revealed that multiple mechanisms for immune protection were induced by immunisation with IC. For instance, our data demonstrated that IC induced a robust antibody response (Table 1) that may confer protection in this model. Using IL-17AKO mice, we demonstrated the important role of IL-17A in immune protection (Fig. 5d–f) and showed that IL-17A contributed to vaccine efficacy. Immunisation with IC still induced protection in the IL-17AKO mice that mainly occurred through the functional antibody response. In conclusion, the high protection level induced by immunisation with IC was mainly dependent on antibody responses and the IL-17A immune pathway.

To date, most vaccines have evaluated the protective effect against a few laboratory strains. However, studies should focus on clinical *S. aureus* isolates because these strains show considerable differences both in the repertoire of surface proteins and sequence variation in their protein binding domains^12^. Immunisation with IC showed broad protection against the WHO2 strain and three clinical *S. aureus* isolates collected from Chongqing, Guangzhou, and Beijing hospitals (Supplemental Table 1). Overall, these results suggested that immunisation with IC was capable of protecting mice against a lethal challenge with different *S. aureus* strains.

In *S. aureus* pneumonia, active immunisation can significantly reduce lung bacterial burdens 1 day post-infection. In the control group, we found that the bacterial burdens continued to decrease over time but were still significantly higher compared with those in the IC group (Fig. 6c). While the severity of pneumonia was controlled in the IC group, the control group worsened over time (Fig. 6c). The most likely explanation for this result is that powerful antibody-mediated bacteria killing in the acute infection phase reduced the bacterial burden in the lung and thereby reduced lung damage. The temperature and weight loss were controlled in the IC group 1 day post-infection, on the contrary, significant temperature and weight loss happened in the AlPO_4_ group (Fig. 6a,b). These results demonstrated that immunisation with IC mainly functioned in the acute infection phase by improving bacterial killing and protecting the lung from damage due to pneumonia. Many studies have shown that vaccines offer protection from pneumonia primarily by inducing antibody production^50^,^51^,^52^. Additionally, some studies have shown that the products of CD4^+^ T cells (i.e., IL-17A) are important in acute pneumonia^53^. In our previous section, we demonstrated that IC could induce strong humoral and cellular responses, both of which may play important roles in defending against *S. aureus* pneumonia.

Recently, Delfani *et al.* had shown that they combined with ClfA, IsdB and gamma hemolysin B (HlgB) fragments and constructed the multi-subunit fusion vaccine ClfA-IsdB-HlgB^54^. Immunisation with ClfA-IsdB-HlgB induced a protective effect in mice sepsis model, and it also induced strong antibody responses. Although ClfA-IsdB-HlgB and IC both contain the fragments of ClfA and IsdB, the truncated domains were different, and may induce different immune protective effects. In our paper, besides sepsis model, we used pneumonia model to evaluate the protective effect of IC, and immunisation with IC also conferred protection in all the five *S. aureus* strains. Thus, immunisation with IC shows broad protective effects in different models and different strains. Meanwhile, we evaluated the functional antibodies through OPA and passive immunisation, and detected Th17 immune pathway played a key role in combating against *S. aureus*.

In our previous research, we designed Hla_H35L_IsdB_348-465_ as an *S. aureus* vaccine candidate based on the reverse vaccinology^30^. In the current study, IC chimeric vaccine was different from Hla_H35L_IsdB_348-465_ in the properties of components, because IsdB and ClfA are both *S. aureus* surface proteins. Meanwhile, the IsdB domains in the IC and Hla_H35L_IsdB_348-465_ were different: IC contained the NEAT1 domain of IsdB, whereas Hla_H35L_IsdB_348-465_ contained the NEAT2 domain. Continuous B cell epitope prediction (http://tools.iedb.org/bcell/) indicated that NEAT1 domain may have advantages in antibody responses. Later, we combined IsdB_151-277_ with ClfA_33-213_, and generated the novel chimeric vaccine IC. Strong antibody responses are the advantages of surface proteins, and interestingly, immunisation with IC also induced strong IL-17A responses. In conclusion, IC can be regarded as a novel *S. aureus* vaccine candidate. When combined with other antigens, such as Hla_H35L_IsdB_348-465_, IC may play a more important role in the fight against *S. aureus* infections.

## Additional Information

**How to cite this article**: Yang, L. *et al.* Protective efficacy of the chimeric *Staphylococcus aureus* vaccine candidate IC in sepsis and pneumonia models. *Sci. Rep.* **6**, 20929; doi: 10.1038/srep20929 (2016).

## Supplementary Material

Supplementary Information

## Figures and Tables

**Table 1:** Antibody responses induced by active immunisation with recombinant proteins.

Recombinant proteins	Antibody response^#^		
	IgG	IgG1	IgG2a
IsdB	1.0733 ± 0.1206	0.7052 ± 0.0430	0.1546 ± 0.0708
ClfA_33-213_	0.6747 ± 0.3884	0.6475 ± 0.0958	0.1348 ± 0.0643
IC	1.3467 ± 0.0443	1.0529 ± 0.0508	0.179 ± 0.10258

^#^Antibody response is expressed as mean absorbance at 450 nm ± SD. All serum samples were diluted 1:6000.

**Table 2:** IgG titers of specific antibodies for recombinant proteins (IsdB and ClfA_33-213_).

Recombinant proteins	Anti-IsdB antibody^*^	Anti-ClfA_33-213_ antibody^*^	Significance^#^
IsdB	85,333 ± 13,492	ND	
ClfA_33-213_	ND	120,000 ± 32,984	
IC	80,000 ± 16,000	128,000 ± 28,621	^*a*^*P* > 0.05, ^*b*^*P* > 0.05

^*^IgG titers (mean serum titers ± SD) in response to immunization of mice as determined by ELISA (n = 6 animals).

^#^Specific antibody titers raised against proteins were not significantly different when immunizing antigens were administered individually or in combination.

^*a*^*P*, anti-IsdB antibodies from the mice immunized with IsdB were compared to those from the mice immunized with IC; ^*b*^*P*, anti-ClfA_33-213_ antibodies from the mice immunized with ClfA_33-213_ were compared with those from the mice immunized with IC.

ND means not detected.

## References

[b1] Lowy F. D. Staphylococcus aureus infections. N Engl J Med 339, 520–32 (1998). 9709046 10.1056/NEJM199808203390806

[b2] Klein E., Smith D. L. & Laxminarayan R. Hospitalizations and deaths caused by methicillin-resistant Staphylococcus aureus, United States, 1999-2005. Emerg Infect Dis 13, 1840–6 (2007). 18258033 10.3201/eid1312.070629 PMC2876761

[b3] Graber C. J. *et al.* Intermediate vancomycin susceptibility in a community-associated MRSA clone. Emerging infectious diseases 13, 491 (2007). 17552110 10.3201/eid1303.060960 PMC2725904

[b4] Chang S. *et al.* Infection with vancomycin-resistant Staphylococcus aureus containing the vanA resistance gene. New England Journal of Medicine 348, 1342–1347 (2003). 12672861 10.1056/NEJMoa025025

[b5] Zhu W. *et al.* Vancomycin-resistant Staphylococcus aureus isolates associated with Inc18-like vanA plasmids in Michigan. Antimicrobial agents and chemotherapy 52, 452–457 (2008). 18056272 10.1128/AAC.00908-07 PMC2224762

[b6] Harro J. M. *et al.* Vaccine development in Staphylococcus aureus: taking the biofilm phenotype into consideration. FEMS Immunol Med Microbiol 59, 306–23 (2010). 20602638 10.1111/j.1574-695X.2010.00708.x PMC2936112

[b7] Schaffer A. C. & Lee J. C. Staphylococcal vaccines and immunotherapies. Infect Dis Clin North Am 23, 153–71 (2009). 19135920 10.1016/j.idc.2008.10.005

[b8] Projan S. J., Nesin M. & Dunman P. M. Staphylococcal vaccines and immunotherapy: to dream the impossible dream? Curr Opin Pharmacol 6, 473–9 (2006). 16870507 10.1016/j.coph.2006.04.005

[b9] Proctor R. A. Is there a future for a Staphylococcus aureus vaccine? Vaccine 30, 2921–7 (2012). 22115633 10.1016/j.vaccine.2011.11.006

[b10] Proctor R. A. Challenges for a universal Staphylococcus aureus vaccine. Clin Infect Dis 54, 1179–86 (2012). 22354924 10.1093/cid/cis033

[b11] Daum R. S. & Spellberg B. Progress toward a Staphylococcus aureus vaccine. Clin Infect Dis 54, 560–7 (2012). 22186773 10.1093/cid/cir828 PMC3404717

[b12] Foster T. J., Geoghegan J. A., Ganesh V. K. & Hook M. Adhesion, invasion and evasion: the many functions of the surface proteins of Staphylococcus aureus. Nat Rev Microbiol 12, 49–62 (2013). 24336184 10.1038/nrmicro3161 PMC5708296

[b13] Kim H. K. *et al.* IsdA and IsdB antibodies protect mice against Staphylococcus aureus abscess formation and lethal challenge. Vaccine 28, 6382–92 (2010). 20226248 10.1016/j.vaccine.2010.02.097 PMC3095377

[b14] Kuklin N. A. *et al.* A novel Staphylococcus aureus vaccine: iron surface determinant B induces rapid antibody responses in rhesus macaques and specific increased survival in a murine S. aureus sepsis model. Infect Immun 74, 2215–23 (2006). 16552052 10.1128/IAI.74.4.2215-2223.2006 PMC1418914

[b15] Joshi A. *et al.* Immunization with Staphylococcus aureus iron regulated surface determinant B (IsdB) confers protection via Th17/IL17 pathway in a murine sepsis model. Hum Vaccin Immunother 8, 336–46 (2012). 22327491 10.4161/hv.18946 PMC3426080

[b16] Josefsson E., Hartford O., O’Brien L., Patti J. M. & Foster T. Protection against experimental Staphylococcus aureus arthritis by vaccination with clumping factor A, a novel virulence determinant. J Infect Dis 184, 1572–80 (2001). 11740733 10.1086/324430

[b17] McDevitt D., Francois P., Vaudaux P. & Foster T. J. Molecular characterization of the clumping factor (fibrinogen receptor) of Staphylococcus aureus. Mol Microbiol 11, 237–48 (1994). 8170386 10.1111/j.1365-2958.1994.tb00304.x

[b18] Moreillon P. *et al.* Role of Staphylococcus aureus coagulase and clumping factor in pathogenesis of experimental endocarditis. Infection and immunity 63, 4738–4743 (1995). 7591130 10.1128/iai.63.12.4738-4743.1995 PMC173679

[b19] Vaudaux P. E. *et al.* Use of adhesion-defective mutants of Staphylococcus aureus to define the role of specific plasma proteins in promoting bacterial adhesion to canine arteriovenous shunts. Infection and immunity 63, 585–590 (1995). 7822026 10.1128/iai.63.2.585-590.1995 PMC173036

[b20] Sullam P. M., Bayer A. S., Foss W. M. & Cheung A. L. Diminished platelet binding *in vitro* by Staphylococcus aureus is associated with reduced virulence in a rabbit model of infective endocarditis. Infection and immunity 64, 4915–4921 (1996). 8945526 10.1128/iai.64.12.4915-4921.1996 PMC174468

[b21] Hall A. E. *et al.* Characterization of a protective monoclonal antibody recognizing Staphylococcus aureus MSCRAMM protein clumping factor A. Infect Immun 71, 6864–70 (2003). 14638774 10.1128/IAI.71.12.6864-6870.2003 PMC308922

[b22] Domanski P. J. *et al.* Characterization of a humanized monoclonal antibody recognizing clumping factor A expressed by Staphylococcus aureus. Infect Immun 73, 5229–32 (2005). 16041045 10.1128/IAI.73.8.5229-5232.2005 PMC1201235

[b23] Narita K. *et al.* Role of interleukin-17A in cell-mediated protection against Staphylococcus aureus infection in mice immunized with the fibrinogen-binding domain of clumping factor A. Infect Immun 78, 4234–42 (2010). 20679443 10.1128/IAI.00447-10 PMC2950370

[b24] Fowler V. G. *et al.* Effect of an investigational vaccine for preventing Staphylococcus aureus infections after cardiothoracic surgery: a randomized trial. JAMA 309, 1368–1378 (2013). 23549582 10.1001/jama.2013.3010

[b25] Patti J. M. A humanized monoclonal antibody targeting Staphylococcus aureus. Vaccine 22 Suppl 1, S39–43 (2004). 15576200 10.1016/j.vaccine.2004.08.015

[b26] Stranger-Jones Y. K., Bae T. & Schneewind O. Vaccine assembly from surface proteins of Staphylococcus aureus. Proc Natl Acad Sci USA 103, 16942–7 (2006). 17075065 10.1073/pnas.0606863103 PMC1636558

[b27] Kim S. T., Chung S. W., Jung J. H., Ha J. S. & Kang I. G. Association of T cells and eosinophils with Staphylococcus aureus exotoxin A and toxic shock syndrome toxin 1 in nasal polyps. Am J Rhinol Allergy 25, 19–24 (2011). 21711967 10.2500/ajra.2011.25.3564

[b28] Anderson A. S. *et al.* Development of a multicomponent Staphylococcus aureus vaccine designed to counter multiple bacterial virulence factors. Hum Vaccin Immunother 8, 1585–94 (2012). 22922765 10.4161/hv.21872 PMC3601133

[b29] Jansen K. U., Girgenti D. Q., Scully I. L. & Anderson A. S. Vaccine review: “Staphyloccocus aureus vaccines: problems and prospects”. Vaccine 31, 2723–30 (2013). 23624095 10.1016/j.vaccine.2013.04.002

[b30] Zuo Q. F. *et al.* Evaluation of the protective immunity of a novel subunit fusion vaccine in a murine model of systemic MRSA infection. PLoS One 8, e81212 (2013). 24324681 10.1371/journal.pone.0081212 PMC3852261

[b31] Uruse M. *et al.* Phase separation of myelin sheath in Triton X-114 solution: predominant localizaion of the 21.5 kDa isoform of myelin basic protein in the lipid-raft-associated domain. Journal of biochemistry 155, 265–271 (2014). 24459152 10.1093/jb/mvu005

[b32] Robertson C. M. *et al.* Neutrophil depletion causes a fatal defect in murine pulmonary Staphylococcus aureus clearance. J Surg Res 150, 278–85 (2008). 18621398 10.1016/j.jss.2008.02.009 PMC2605623

[b33] Burton R. L. & Nahm M. H. Development of a fourfold multiplexed opsonophagocytosis assay for pneumococcal antibodies against additional serotypes and discovery of serological subtypes in Streptococcus pneumoniae serotype 20. Clinical and Vaccine Immunology 19, 835–841 (2012). 22518015 10.1128/CVI.00086-12 PMC3370448

[b34] Cooke R. P. & Jenkins C. T. Comparison of commercial slide agglutination kits with a tube coagulase test for the rapid identification of Staphylococcus aureus from blood culture. J Clin Pathol 50, 164–6 (1997). 9155701 10.1136/jcp.50.2.164 PMC499745

[b35] Luijendijk A., van Belkum A., Verbrugh H. & Kluytmans J. Comparison of five tests for identification of Staphylococcus aureus from clinical samples. J Clin Microbiol 34, 2267–9 (1996). 8862596 10.1128/jcm.34.9.2267-2269.1996 PMC229229

[b36] Grigg J. C., Vermeiren C. L., Heinrichs D. E. & Murphy M. E. Haem recognition by a Staphylococcus aureus NEAT domain. Mol Microbiol 63, 139–49 (2007). 17229211 10.1111/j.1365-2958.2006.05502.x

[b37] Pilpa R. M. *et al.* Functionally distinct NEAT (NEAr Transporter) domains within the Staphylococcus aureus IsdH/HarA protein extract heme from methemoglobin. J Biol Chem 284, 1166–76 (2009). 18984582 10.1074/jbc.M806007200 PMC2613621

[b38] Aoki W. *et al.* Design of a novel antimicrobial peptide activated by virulent proteases. Chem Biol Drug Des 80, 725–33 (2012). 22863111 10.1111/cbdd.12012

[b39] Fritz S. A. *et al.* A serologic correlate of protective immunity against community-onset Staphylococcus aureus infection. Clin Infect Dis 56, 1554–61 (2013). 23446627 10.1093/cid/cit123 PMC3641868

[b40] Ebert T. *et al.* A fully human monoclonal antibody to Staphylococcus aureus iron regulated surface determinant B (IsdB) with functional activity *in vitro* and *in vivo*. Hum Antibodies 19, 113–28 (2010). 21178283 10.3233/HAB-2010-0235

[b41] Pancari G. *et al.* Characterization of the mechanism of protection mediated by CS-D7, a monoclonal antibody to Staphylococcus aureus iron regulated surface determinant B (IsdB). Front Cell Infect Microbiol 2, 36 (2012). 22919628 10.3389/fcimb.2012.00036 PMC3417506

[b42] Brown M. *et al.* Selection and characterization of murine monoclonal antibodies to Staphylococcus aureus iron-regulated surface determinant B with functional activity *in vitro* and *in vivo*. Clin Vaccine Immunol 16, 1095–104 (2009). 19553551 10.1128/CVI.00085-09 PMC2725548

[b43] Dryla A. *et al.* Comparison of antibody repertoires against Staphylococcus aureus in healthy individuals and in acutely infected patients. Clin Diagn Lab Immunol 12, 387–98 (2005). 15753252 10.1128/CDLI.12.3.387-398.2005 PMC1065207

[b44] Casadevall A. & Pirofski L. A. Antibody-mediated regulation of cellular immunity and the inflammatory response. Trends Immunol 24, 474–8 (2003). 12967670 10.1016/s1471-4906(03)00228-x

[b45] Stockinger B. & Veldhoen M. Differentiation and function of Th17 T cells. Curr Opin Immunol 19, 281–6 (2007). 17433650 10.1016/j.coi.2007.04.005

[b46] Ellis T. N. & Beaman B. L. Interferon-gamma activation of polymorphonuclear neutrophil function. Immunology 112, 2–12 (2004). 15096178 10.1111/j.1365-2567.2004.01849.x PMC1782470

[b47] Romagnani S., Maggi E., Liotta F., Cosmi L. & Annunziato F. Properties and origin of human Th17 cells. Mol Immunol 47, 3–7 (2009). 19193443 10.1016/j.molimm.2008.12.019

[b48] Yu L. *et al.* Improved protective efficacy of a chimeric Staphylococcus aureus vaccine candidate iron-regulated surface determinant B (N 126- P 361) -target of RNAIII activating protein in mice. Microbiol Immunol 57, 857–64 (2013). 24117875 10.1111/1348-0421.12106

[b49] Spellberg B. & Daum R. Development of a vaccine against Staphylococcus aureus. Semin Immunopathol 34, 335–48 (2012). 22080194 10.1007/s00281-011-0293-5 PMC4184131

[b50] Ragle B. E. & Bubeck Wardenburg J. Anti-alpha-hemolysin monoclonal antibodies mediate protection against Staphylococcus aureus pneumonia. Infect Immun 77, 2712–8 (2009). 19380475 10.1128/IAI.00115-09 PMC2708543

[b51] Bubeck Wardenburg J. & Schneewind O. Vaccine protection against Staphylococcus aureus pneumonia. J Exp Med 205, 287–94 (2008). 18268041 10.1084/jem.20072208 PMC2271014

[b52] Adhikari R. P. *et al.* Novel structurally designed vaccine for S. aureus alpha-hemolysin: protection against bacteremia and pneumonia. PLoS One 7, e38567 (2012). 22701668 10.1371/journal.pone.0038567 PMC3368876

[b53] Kudva A. *et al.* Influenza A inhibits Th17-mediated host defense against bacterial pneumonia in mice. J Immunol 186, 1666–74 (2011). 21178015 10.4049/jimmunol.1002194 PMC4275066

[b54] Delfani S., Mohabati Mobarez A., Imani Fooladi A. A., Amani J. & Emaneini M. Protection of mice against Staphylococcus aureus infection by a recombinant protein ClfA-IsdB-Hlg as a vaccine candidate. Med Microbiol Immunol, doi: 10.1007/s00430-015-0425-y (2015). 26155981

