# Supplementary material for: Protective efficacy of the chimeric Staphylococcus aureus vaccine candidate IC in sepsis and pneumonia models
Source: Sci Rep. 2016 Feb 11;6:20929. doi: 10.1038/srep20929 (PMC4750066; doi:10.1038/srep20929)
Supplement: Supplementary Information [file srep20929-s1.pdf]

Protective efficacy of the chimeric *Staphylococcus aureus* vaccine candidate IC in sepsis and pneumonia models

Liuyang Yang[1], Changzhi Cai[1], Qiang Feng[2], Yun Shi[1], Qianfei Zuo[1], Huijie Yang[1], Haiming Jing[1], Chao Wei[1], Yuan Zhuang[1], Quanming Zou[1*] & Hao Zeng[1*].

[1]National Engineering Research Center of Immunological Products & Department of Microbiology and Biochemical Pharmacy, College of Pharmacy, Third Military Medical University, Chongqing 400038, PR China,

[2] Department of Biological and Chemical Engineering, Chongqing University of Education,Chongqing 400067, PR China.

[*] Corresponding author: Hao Zeng (zeng1109@163.com) and Quanming Zou (qmzou2007@163.com), at National Engineering Research Center of Immunological Products, Department of Microbiology and Biochemical Pharmacy, College of Pharmacy, Third Military Medical University, Chongqing 400038, PR China. Phone: 86-023-68752377. Fax: 086-023-68752377.

Supplemental Table 1. *S. aureus* strains used for the murine sepsis and pneumonia models.

Name	Strain source	Sex	Age	Sample Source	IsdB	ClfA	nuc	mecA	MIC (μg/ml)	Model	Lethal dose
MRSA252	ATCC: BAA-1720				+	+	+	+	256	Sepsis and pneumonia	1×10^9
WHO 2	Third Military Medical University				+	+	+	+	256	sepsis model	5×10^8
BJ03	Beijing 263 hospital	male	31	wound	+	+	+	-	256	sepsis model	3×10^8
CQ19	Chongqing southwest hospital	female	22	blood	+	+	+	+	256	sepsis model	3×10^8
GZ02	Guangzhou south hospital	male	21	punctate	+	+	+	+	12	sepsis model	3×10^8

Supplemental Table 2. Primers used for construction of IsdB$_{151\text{-}277}$ClfA$_{33\text{-}213}$

P1	GCGGATCCATGGGCAGCGCACCAAACTCTCGTCCA
P2	AACATCAACCGGAACGTATTCAGTTTTGAATTTATC
P3	TACGTTCCGGTTGATGTTAGCAGTAAAGAAGCAGAT
P4	GCGGCCGCTTATCACTCGAGCATACGAGGCGCACT

Supplemental Figure 1. The exterior of lungs in IC group and $AlPO_4$ group.

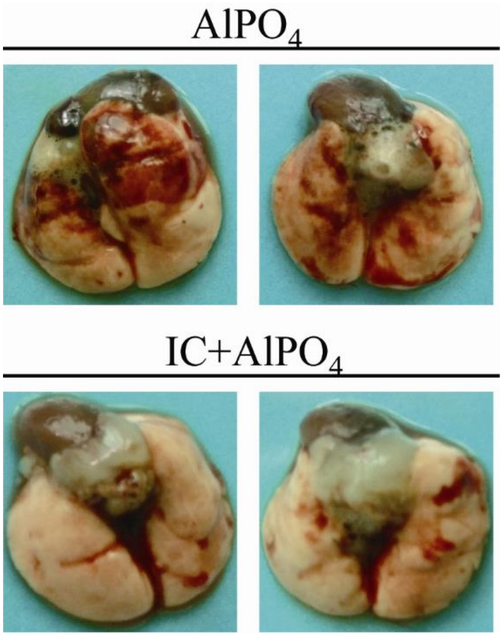

C57 mice were immunised as described before. Then, mice were inoculated with 4×10^8 CFUs of MRSA252 suspension in the naris. One day after infection, lungs were collected and washed by PBS.
